# Evaluation of Recombinant Kpkt Cytotoxicity on HaCaT Cells: Further Steps towards the Biotechnological Exploitation Yeast Killer Toxins

**DOI:** 10.3390/foods10030556

**Published:** 2021-03-08

**Authors:** Gavino Carboni, Ivana Marova, Giacomo Zara, Severino Zara, Marilena Budroni, Ilaria Mannazzu

**Affiliations:** 1 Department of Agricultural Sciences, University of Sassari, Viale Italia 39, 07100 Sassari, Italy; g.carboni43@studenti.uniss.it (G.C.); gzara@uniss.it (G.Z.); szara@uniss.it (S.Z.); mbudroni@uniss.it (M.B.); 2 Faculty of Chemistry, Brno University of Technology, Purkyňova 464/118, Královo Pole, 61200 Brno, Czech Republic

**Keywords:** Kpkt, yeast killer toxin, natural antimicrobial, HaCaT cells

## Abstract

The soil yeast *Tetrapisispora phaffii* secretes a killer toxin, named Kpkt, that shows β-glucanase activity and is lethal to wine spoilage yeasts belonging to *Kloeckera/Hanseniaspora*, *Saccharomycodes* and *Zygosaccharomyces*. When expressed in *Komagataella phaffii*, recombinant Kpkt displays a wider spectrum of action as compared to its native counterpart, being active on a vast array of wine yeasts and food-related bacteria. Here, to gather information on recombinant Kpkt cytotoxicity, lyophilized preparations of this toxin (LrKpkt) were obtained and tested on immortalized human keratinocyte HaCaT cells, a model for the stratified squamous epithelium of the oral cavity and esophagus. LrKpkt proved harmless to HaCaT cells at concentrations up to 36 AU/mL, which are largely above those required to kill food-related yeasts and bacteria in vitro (0.25–2 AU/mL). At higher concentrations, it showed a dose dependent effect that was comparable to that of the negative control and therefore could be ascribed to compounds, other than the toxin, occurring in the lyophilized preparations. Considering the dearth of studies regarding the effects of yeast killer toxins on human cell lines, these results represent a first mandatory step towards the evaluation the possible risks associated to human intake. Moreover, in accordance with that observed on *Ceratitis capitata* and *Musca domestica*, they support the lack of toxicity of this toxin on non-target eukaryotic models and corroborate the possible exploitation of killer toxins as natural antimicrobials in the food and beverages industries.

## 1. Introduction

Yeast killer toxins are proteins, often glycoproteins, which recognize specific receptors on the surface of their sensitive targets and kill them through different modes of action. According to Klassen et al. [1] about 100 yeast killer species have been described so far. The spectrum of action of yeast killer toxins covers spoilage microorganisms significant for the fermentative [2,3,4,5,6,7,8,9,10,11,12] and food and feed industries [13,14], but it also encompasses microbial pathogens of clinical interest [15,16,17,18], and plant pathogens [19,20,21]. Thus, yeast killer toxins have a wide range of possible applications as natural antimicrobials in the agri-food industry, as well as therapeutic agents for animal and human infections and for biological control of plant pathogens [22,23,24]. However, although a number of inventions regarding killer toxins application have been patented so far, their exploitation at the industrial level never took off [24]. The wine sector represents an exception in this sense. Here, the direct inoculation of yeast killer strains is admitted and recommended to ensure the dominance of the inoculated starter [5] and numerous commercial starters for oenology are killer strains of *S. cerevisiae.* Indeed, the effectiveness of *S. cerevisiae* killer strains in wine depends on several factors as reported by [5] and their dominance is not obvious also considering the narrowness of their spectrum of action. On the contrary non-*Saccharomyces* yeast killer toxins have a broader spectrum of action and some of them are active on spoilage yeasts of relevance for the wine industry [24,25].

One of these toxins, naturally secreted by the yeast *Tetrapisispora phaffii* and known as Kpkt, is effective on wine yeasts ascribed to the genera *Kloeckera/Hanseniaspora*, *Saccharomycodes,* and *Zygosaccharomyces* [4]. Further works showed that Kpkt interacts with β-1,3 and β-1,6 branched glucans [7], and that it affects the ultrastructure of the cell wall of the sensitive yeasts due to its β-glucanase activity [7,26]. Accordingly, Tp*BGL2* gene, that codes for Kpkt, shows more than 75% identity with β-1,3-glucanase encoding genes of other yeasts species and *T. phaffii* loses killer and β-glucanase activities following Tp*BGL2* gene deletion [27]. Interestingly, Kpkt retains killer activity under winemaking conditions for up to two weeks [28], thus suggesting its possible exploitation in the wine industry in place of SO_2_ [28]. Indeed, *K. phaffii* DBVPG 6076 is a soil isolate, not suitable for direct inoculation in grape must. Moreover, it secretes finite amounts of Kpkt thus hampering the large scale production of the toxin in view of its direct utilization in the wine industry. For that, the heterologous production of Kpkt was obtained in *Komagataella phaffii* GS115 (formerly *Pichia pastoris*) [29]. This is a GRAS host for the production of heterologous proteins that find application in the food and biopharmaceutical industries [30]. More recently, Carboni et al. [18] produced a lyophilized preparation of recombinant Kpkt that displays killer activity on a vast array of wine yeasts, among which *Dekkera/Brettanomyces* sp., but also on Gram positive (*Lactobacillus rhamnosus*, *Lactobacillus plantarum, Staphylococcus aureus, Listeria monocytogenes*) and Gram-negative bacteria (*Salmonella bongori,* and *Escherichia coli*) [18]. Thus, the lower specificity of action of recombinant Kpkt (rKpkt), as compared to native Kpkt, and its availability in the form of a manageable ready-to-use compound, suggest a plethora of possible applications for rKpkt in the wine, sweet beverage and more in general the food industries.

Indeed, similar to other putative natural antimicrobials, yeast killer toxins suffer of the dearth of information regarding their toxicity on non-target eukaryotic models. According to Pfeiffer et al. [31] no effect of *S. cerevisiae* killer toxin was observed on animal model systems. Similarly, recombinant and native Kpkt proved harmless on *Ceratitis capitata* and *Musca domestica*, two insect species recently utilized as non-mammalian eukaryotic models [18]. Conversely, Pettoello-Mantovani et al. [32], hypothesized that the killer toxin produced by *Hansenula anomala* is involved in the onset of *H. anomala*-induced enteritis.

Here, in order to gather information on recombinant Kpkt cytotoxicity, the effect of a lyophilized preparation of rKpkt was evaluated on human keratinocyte HaCaT cells, a model for the stratified squamous epithelium of the oral cavity and esophagus [33]. Particularly, keratinocytes are exposed to molecules present in ingested foods and have already been used to assess the cytotoxicity of antimicrobials among which nisin, chitosan, bronopol, chlorhexidine, and others [34,35].

## 2. Materials and Methods

### 2.1. Microorganisms, Cloning Vectors, and Growth Media

Microorganisms used are reported in Table 1. Recombinant clone 17 (rc#17) was obtained by transforming *K. phaffii* GS115 with pPIC9TpIMHisTag. This vector, provided by DNA 2.0 (Menlo Park, CA, USA), contains a codon optimized Kpkt coding sequence (TpIM) under the control of *AOX*1 promoter and downstream *S. cerevisiae* α leader sequence of secretion [29], and carries a HisTag in C terminus. Transformation and screening of transformants were carried out as already described [29].

Culture media were the following. YEPD: 2% glucose, 1% yeast extract and 2% peptone; BMGY: 1% glycerol (*w/v*), 1% yeast extract, 2% peptone, 1.34% YNB w/o aminoacids and 0.00004% biotin; BMGluY: as BMGY with 1% glucose instead of glycerol; BMMY: 0.5 or 1% methanol (*v/v*), 1% yeast extract, 2% peptone, 1.34% YNB w/o aminoacids and 0.00004% biotin. All media were buffered at pH 4.5 with citrate-phosphate buffer (0.1 M citric acid, 0.2 M Na_2_HPO_4_).

Yeasts were kept on YEPD at 4 °C and in YEPDgly (YEPD with addition of 40% glycerol) at −80 °C for short and long-term storage, respectively.

### 2.2. Production of Native and Recombinant Kpkt in Flask and Bioreactor

Baffled flask production of recombinant Kpkt (rKpkt) and bioreactor productions of native and recombinant Kpkt were carried out as already described [18] with significant modifications. In particular, for rc#17 and rc#24 bioreactor cultivation, two methanol feeding strategies were utilized in methanol fed batch phase: the dissolved oxygen (DO) strategy and methanol continuous feeding. According to the DO strategy, methanol feeding rate is in cascade with the dissolved oxygen level. For methanol continuous feeding, methanol feeding rate is taken to ~7.3 mL/L/h after 1 h and to ~10.9 mL/L/h after 2 more h. Methanol fed batch phase lasted approximately 72 h after which the culture broth was harvested by centrifugation (5000× *g* for 10 min) and the cell-free supernatant was subjected to micro-filtration (0.45 µm filter Minisart, Sartorius, Göttingen, Germany) and well plate assay, as already reported [29].

### 2.3. Cell-Free Supernatant Ultrafiltration and Lyophilization

Cell-free supernatants of rc#17, rc#24, and *T. phaffii* DBVPG6076 were concentrated by means of a TFF/Cross Flow System (Repligen, Waltham, MA, USA) with a 10 KDa cut-off membrane and 60-fold concentrated samples were lyophilized as already described [18]. The lyophilized preparations were solubilized in sterile distilled water (100 mg/mL) and total protein content was measured according to Bradford [36] while killer toxin concentration was evaluated by well plate assay.

### 2.4. Well Plate Assay for the Evaluation of Killer Activity and the Determination of Killer Toxin Concentration

One hundred μL of a cell suspension of the sensitive strain in sterile distilled water (OD_600_ 0.1–0.2) was distributed on YEPD plates and 100 μL aliquots of the sample to be tested were filled into wells cut into the plate. Killer activity was recorded based on the appearance of an inhibition halo around the well after 3 days of incubation at 25 °C. rc#24 was used as the negative control of killer activity. Killer toxin concentration was expressed as arbitrary units (AU) [4]. Briefly: 10, 15, 25, 50, and 75 µL of cell-free supernatant taken to 100 µL with citrate phosphate buffer (pH 4.5), and 100 µL of cell-free supernatant, were subjected to well plate assay. The diameters of the inhibition halos around each well were measured (mm) after 3 days of incubation at 25 °C. The Log of toxin concentration and the diameters of the inhibition halos were reported on the abscissa and on the ordinate of a Cartesian axes system, respectively. Given the linear relationship between the Log of toxin concentrations and the diameters of the inhibition halos, it was established that 1 AU corresponds to the toxin concentration in 100 μL that results in an inhibition halo of 20 mm (included the diameter of the well).

### 2.5. Evaluation of β-Glucanase Activity

β-glucanase activity was evaluated according to Notario [37]. Briefly, 800 μL of 0.25% laminarin solubilized in 50 mM sodium acetate (pH 5.0, with glacial acetic acid) was added to 200 μL of the sample to be tested. After 30 min incubation at 37 °C the mixtures were boiled for 5 min to stop the reaction. Laminarinase (Sigma-Aldrich, St. Louis, MO, USA) (0.01 U in 1-mL reaction mixture) was utilized as positive control of activity. Negative controls of enzymatic activity for LrKpkt and LnKpkt were lyophilized preparations of 60-fold concentrated cell-free supernatant of rc#24 and heat-treated cell-free supernatant of *T. phaffii* DBVPG 6076, respectively. GAGO-20 kit (Sigma-Aldrich) was utilized to quantify the glucose released. Results presented are means ± standard deviation of at least three technical replicates of three independent experiments.

### 2.6. Human Cell Line Toxicity Test

HaCaT cells were grown as a monolayer at 37 °C, under 5% CO_2_ on Dulbecco’s Modified Eagle Medium (DMEM, Sigma Aldrich) liquid medium (pH 7.4) supplemented with 2 mM l-glutamine, 10% fetal bovine serum (FBS) and penicillin-streptomycin (100 U/mL–0.1 mg/mL). After 3 days, 3 × 10^4^ cells/mL were aliquoted in 96-well plates and exposed to increasing concentrations of LrKpkt and LnKpkt (ranging from 0 to 72 AU). Lyophilized preparations of rc#24 and heat inactivated 60-fold concentrated cell-free supernatant of *T. phaffii* DBVPG 6076 were utilized as negative controls for LrKpkt and LnKpkt, respectively. After 24 h of incubation with the toxin, cells were observed under a bright field microscope to gather preliminary information on cell viability. Cell viability was determined by MTT [3-(4,5-dimethylthiazol-2-yl)-2,5-diphenyltetrazolium (MTT) bromide] test (MTT Cell Growth Assay Kit, Sigma Aldrich). After 3 h incubation in MTT, 10% SDS was added to each well and OD_562_ was recorded to evaluate the percentage of viable cells in respect to the negative control. Results presented are mean ± standard deviation of at least three technical replicates of three independent experiments.

### 2.7. Data Analysis

Data were subjected to one-way analysis of variance (ANOVA). Tukey test was employed for post-hoc comparisons and the critical value for significance level (*p*) was set at 0.05. All statistical analyses were performed using R-studio for Windows, version 10 (RStudio, PBC, Boston, MA, USA).

## 3. Results and Discussion

### 3.1. Scale up of Recombinant Kpkt Production

In previous works the heterologous production of the yeast killer toxin Kpkt in *K. phaffii* was obtained by transforming GS115 with vector pPIC9TpIM [18,29]. Here, considering that the utilization of Tag containing expression vectors may facilitate recombinant protein purification, pPIC9TpIMHisTag was utilized. The only recombinant clone showing killer activity among the 72 transformants that integrated the expression cassette (rc#17), was grown in bioreactor to obtain the production of rKpkt. Similar to that reported for rc#6 [18], fed batch bioreactor cultivation of rc#17 was organized into three phases. In particular, rc#17 was fed glucose or glycerol during the first two phases to obtain a high cell density and methanol during the third phase in order to induce the expression of Kpkt coding sequence under the control of *AOX*1 promoter [18,38,39,40,41,42,43]. Since both glucose and glycerol repress *AOX*1 promoter, no toxin production was detected on the two carbon sources during the first two phases of cultivation. However, rc#17 took on average 48 h on glucose and about 96 h on glycerol to reach the requested cell density (OD_600_ 350–400). Thus, contrary to that observed for rc#6 [18], glucose proved better than glycerol in increasing biomass production rate prior to methanol induction. Based on that, although no differences in the amount of toxin produced at the end of the fermentation process were observed, glucose feeding was utilized for the first two stages. Regarding methanol feeding, since high concentrations of methanol are toxic to the yeast cells, besides the continuous feeding strategy, here also the dissolved oxygen (DO) strategy was applied. However, while no rKpkt production was observed when the DO strategy was utilized, methanol continuous feeding resulted in a time dependent accumulation of rKpkt in the supernatant. In particular, the cell-free supernatant of rc#17 contained 1.8 ± 0.04, 7.3 ± 0.02 and 14.1 ± 0.15 AU/mL of recombinant killer toxin after 24, 48 and 70 h, respectively. Interestingly the final concentration of rKpkt obtained in bioreactor was significantly higher (*p* < 0.05) than that obtained after 120 h in baffled flask (11.0 ± 0.08 AU/mL). Bioreactor cultivation of *T. phaffii* DBVPG 6076 led to the production of 7.9 ± 0.09 AU/mL of native Kpkt (nKpkt). This concentration was significantly lower than that obtained with rc#17 (*p* < 0.001), thus confirming that heterologous expression of KpKt is crucial to increase Kpkt production.

### 3.2. Production of Lyophilized Preparations of Kpkt and Evaluation of Killer and β-Glucanase Activities

**Figure 1:**
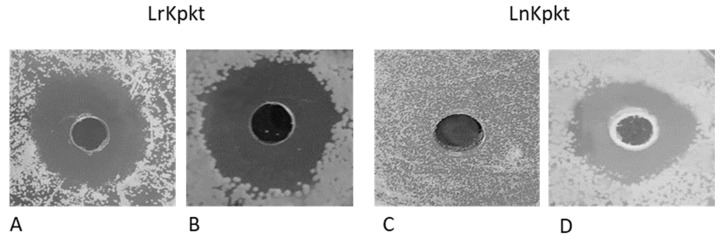
Well plate assay of lyophilized preparations of recombinant (LrKpkt) and native (LnKpkt) Kpkt. *D. bruxellensis* DiSVA 692 (**A**,**C**) and *S. cerevisiae* DBVPG 6500 (**B**,**D**) were utilized as sensitive strains.

Similar to that reported by Carboni et al. [18], following the lyophilization of 40 mL of 60-fold concentrated cell-free supernatant of rc#17 and *T. phaffii* DBVPG 6076, the ready-to-use preparations of recombinant (LrKpkt) and native (LnKpkt) killer toxin, respectively, were produced. In particular, 3.46 g of LrKpkt containing 411 AU/g and 3.78 g of LnKpkt containing 353 AU/g were obtained. LrKpkt and LnKpkt confirmed differences in their spectrum of action [18,29] with LrKpkt being active also on *D. bruxellensis* (Figure 1).

**Figure 2:**
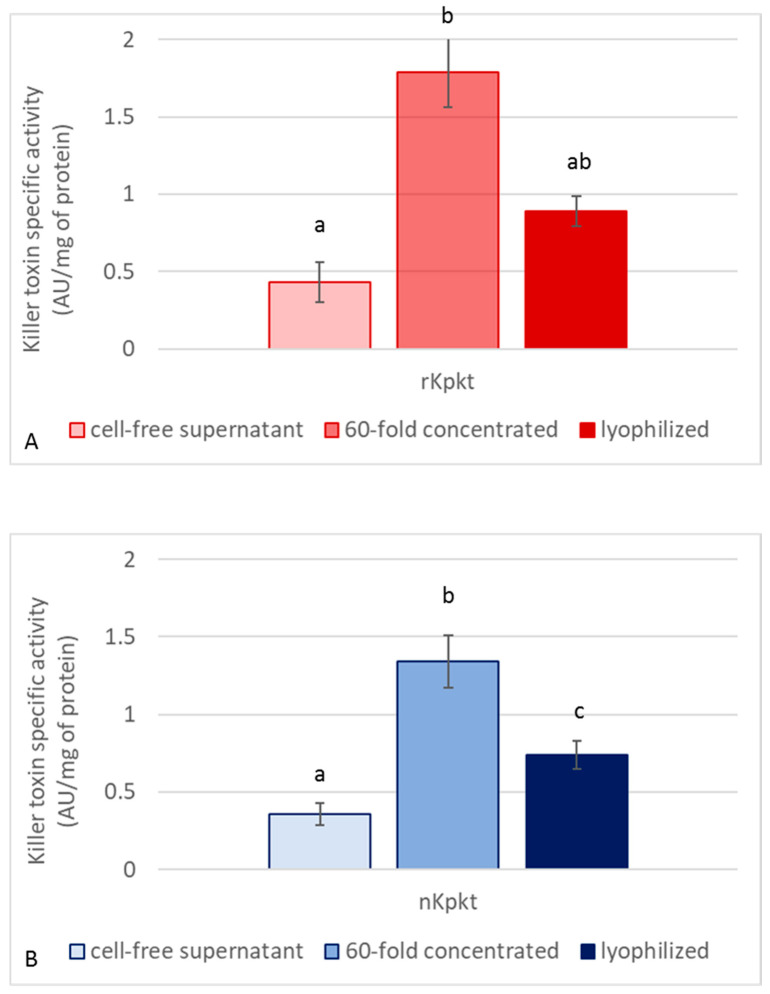
Specific killer activity of recombinant (**A**) and native (**B**) Kpkt. Killer toxin specific activity is expressed as AU/mg of protein. Results are means ± standard deviations of at least three independent experiments. Different letters indicate significantly different results (*p* < 0.001).

**Figure 3:**
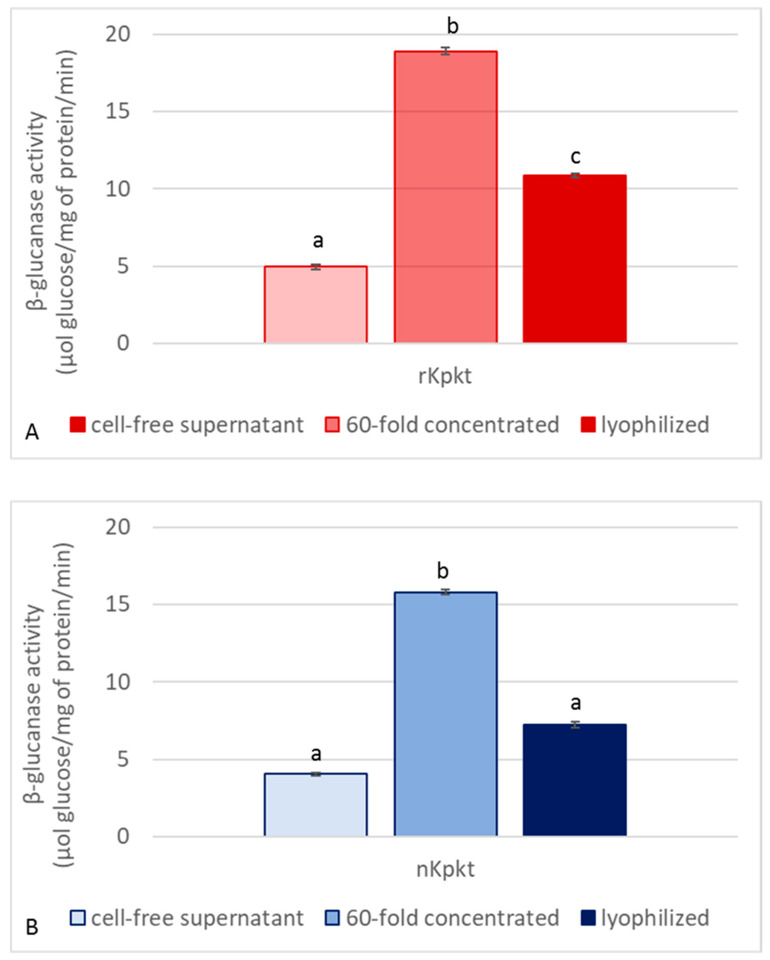
β-glucanase activity of recombinant (**A**) and native (**B**) Kpkt. β-glucanase activity is expressed as μmol of glucose/mg protein/min. Results are means ± standard deviations of at least three independent experiments. Different letters indicate significantly different results (*p* < 0.001).

Since Kpkt hydrolyses β-glucans, and considering that killer activity on yeast sensitive targets is clearly mediated by β-glucanase activity [26], both killer and enzymatic activities of the cell free supernatant, and of the 60-fold concentrated samples, were measured before and after lyophilization. Killer activities of native and recombinant Kpkt preparations were evaluated by means of the well plate assay. As reported in Figure 2, while the 60-fold concentrated samples showed higher killer activity due to the concentration of proteins with MW higher than 10 kDa, among which Kpkt, lyophilization resulted in the decrease of specific killer activity. Regarding β-glucanase activity, the cell free supernatants of rc#17 and *T. phaffii* DBVPG 6076 hydrolyzed laminarin, with specific activities of 4.04 *±* 0.08 and 4.94 *±* 0.15 μmol of glucose/mg protein/min, respectively, that were significantly (*p* < 0.001) higher than that of pure commercial β-glucanase (2.8 *±* 0.11 μmol of glucose/mg protein/min). Indeed, similar to that observed for killer activity, β-glucanase activity showed a considerable increase in the 60-fold concentrated sample while significantly decreasing following lyophilization (*p* < 0.001) (Figure 3), in accordance with the interdependence of the two activities.

### 3.3. Cytotoxicity of rKpkt and nKpkt on HaCaT Cell Lines

**Figure 4:**
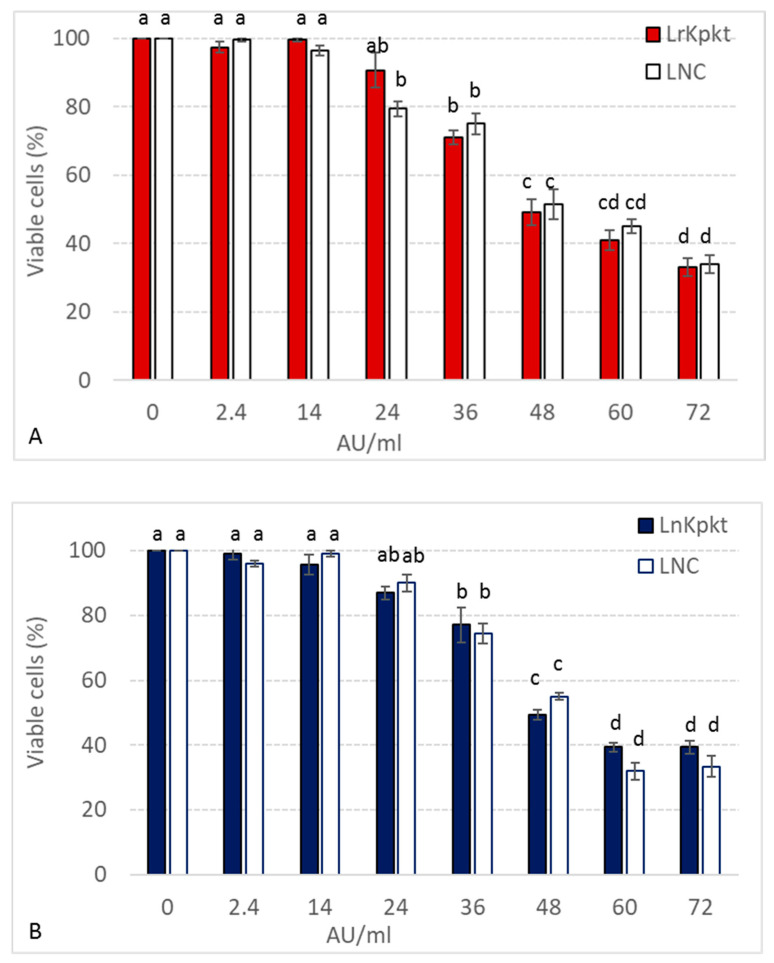
Viability of HaCaT cells after 24 h exposure to increasing concentrations of LrKpkt (**A**) and LnKpkt (**B**). Cell viability was determined in respect to positive controls (0 AU of LrKpkt or LnKpkt). LNC negative controls of killer activity. Results are means ± standard deviation of at least three independent experiments. Same superscript letters indicate results not significantly different (*p* < 0.05).

In spite of the observed reduction in killer and β-glucanase activities, lyophilized preparations are characterized by longer shelf life and ease of management in respect to concentrated cell-free preparations [18]. For these reason LrKpkt and LnKpkt were utilized on human keratinocyte HaCaT cells. Lyophilized recombinant Kpkt, although proving killer on yeast targets, showed no significant effects on human keratinocytes HaCaT cells for concentrations up to 14 AU/mL (Figure 4). The main function of keratinocytes is to protect the inner environment from foreign pathogens and chemical agents, and to signal injury to the host. Thus, in accordance with the suggested safety of yeast killer toxin on animal systems [31], these results are compatible with the hypothesis that LrKpkt, at these concentrations, may not exert a potential health risk for human consumption. Higher concentrations of lyophilized preparations of killer toxins determined a dose dependent effect on HaCaT cells. However, residual cell viability was still beyond 60% for LrKpkt concentrations up to 36 AU/mL (Figure 4) that are largely above those requested to kill food-related yeasts and bacteria in vitro (ranging from 0.25 to 2 AU/mL) [18]. Moreover, no significant differences were observed between LrKpkt and its relevant negative control (LNC). Thus, the dose dependent decrease in residual cell viability could be due to compounds, either culture media components or yeast-produced compounds, other than the toxin, occurring in the lyophilized preparations and toxic to HaCaT cells. Remarkably, LrKpkt effect on HaCaT cells was comparable to that of LKpkt. Thus, despite the lower specificity of rKpkt spectrum of action, LrKpkt and LnKpkt showed no significant difference in the effect exerted on human cells.

## 4. Conclusions

While previous works proved the feasibility of recombinant Kpkt production in *K. phaffii* and the advantages of rKpkt lyophilization, the aim of this work was to gather information on recombinant Kpkt toxicity on human cells. In line with the evidence that each recombinant clone requires a customized set up of culture conditions, a specific protocol was tailored on rc#17 for the production of rKpkt. After having shown that recombinant Kpkt produced by rc#17 maintains its biological functions upon lyophilization, its effect on immortalized human keratinocyte HaCaT cells was tested and compared to that of its native counterpart. In vitro evaluation of native and recombinant Kpkt toxicity on HaCaT cells proved that both are harmless to this cell line at concentrations that are largely above those required to kill food-related yeasts and bacteria in vitro. In accordance with that already observed on *Ceratitis capitata* and *Musca domestica*, these results support the lack of toxicity of Kpkt on non-target eukaryotic models and corroborate the possible exploitation of yeast killer toxins as natural antimicrobials in the food and beverages industries. Moreover, considering the dearth of studies regarding the effects of yeast killer toxins on human cell lines, these results represent a first step towards the evaluation of the possible risks associated to human intake.

## Figures and Tables

**Table 1:** Microbial strains utilized in the present work.

Strain	Source	Characteristic
*Tetrapisispora phaffii* 6076	DBVPG	Native producer of Kpkt
*Komagataella phaffii* GS115	Invitrogen	Host for heterologous expression, *his4*
*Saccharomyces cerevisiae* 6500	DBVPG	Sensitive to Kpkt and rKpkt
*Dekkera bruxellensis* 692	DiSVA	Sensitive to rKpkt
*Komagataella phaffii* rc#24	UNISS	negative control of killer activity [29]
*Komagataella phaffii* rc#17	This study	pPIC9TpIMHisTag in GS115

UNISS: Microbial Culture Collection, Department of Agricultural Sciences, University of Sassari, Sassari, Italy; DiSVA: Culture Collection of Department of Life and Environmental Sciences, Polytechnic University of Marche, Ancona, Italy. DBVPG; Industrial Yeast Collection DBVPG, University of Perugia, Perugia, Italy.
